# Placental mTOR Signaling and Sexual Dimorphism in Metabolic Health across the Lifespan of Offspring

**DOI:** 10.3390/children8110970

**Published:** 2021-10-26

**Authors:** Megan Beetch, Emilyn U. Alejandro

**Affiliations:** Department of Integrative Biology and Physiology, University of Minnesota, Minneapolis, MN 55455, USA; beet0013@umn.edu

**Keywords:** mTOR signaling, placenta, pregnancy, fetal growth restriction, birth weight, obesity, insulin sensitivity, type II diabetes

## Abstract

Robust evidence of fetal programming of adult disease has surfaced in the last several decades. Human and preclinical investigations of intrauterine insults report perturbations in placental nutrient sensing by the mechanistic target of rapamycin (mTOR). This review focuses on pregnancy complications associated with placental mTOR regulation, such as fetal growth restriction (FGR), fetal overgrowth, gestational diabetes mellitus (GDM), polycystic ovarian syndrome (PCOS), maternal nutrient restriction (MNR), preeclampsia (PE), maternal smoking, and related effects on offspring birthweight. The link between mTOR-associated birthweight outcomes and offspring metabolic health trajectory with a focus on sexual dimorphism are discussed. Both human physiology and animal models are summarized to facilitate in depth understanding. GDM, PCOS and fetal overgrowth are associated with increased placental mTOR, whereas FGR, MNR and maternal smoking are linked to decreased placental mTOR activity. Generally, birth weight is reduced in complications with decreased mTOR (i.e., FGR, MNR, maternal smoking) and higher with increased mTOR (GDM, PCOS). Offspring display obesity or a higher body mass index in childhood and adulthood, impaired glucose and insulin tolerance in adulthood, and deficiencies in pancreatic beta-cell mass and function compared to offspring from uncomplicated pregnancies. Defining causal players in the fetal programming of offspring metabolic health across the lifespan will aid in stopping the vicious cycle of obesity and type II diabetes.

## 1. Introduction

Decades of research have revealed that many diseases diagnosed over a lifetime have fetal origins. As a response to various exposures during the developmental period, such as maternal over/under-nutrition, chemicals, and stresses, fetal organs may be mal-programmed due to their inherent plasticity during development. The concept of fetal programming suggests that nutritional and environmental events that occur during critical periods of pregnancy cause permanent effects on the fetus and the long-term health trajectory of the offspring. A robust and important area of research investigating the developmental origins of health and disease (DOHAD) hypothesis has made huge strides in understanding the fetal programming of adult chronic and non-communicable diseases. Myriad epidemiological studies provide evidence of this association as it relates to risk for obesity, hypertension, cardiovascular disease, and diabetes [1,2]. However, mechanism-driven studies, especially those investigating the placenta, which sits at the interface of mother and fetus, comprise an unresolved and understudied area of research.

**Figure 1:**
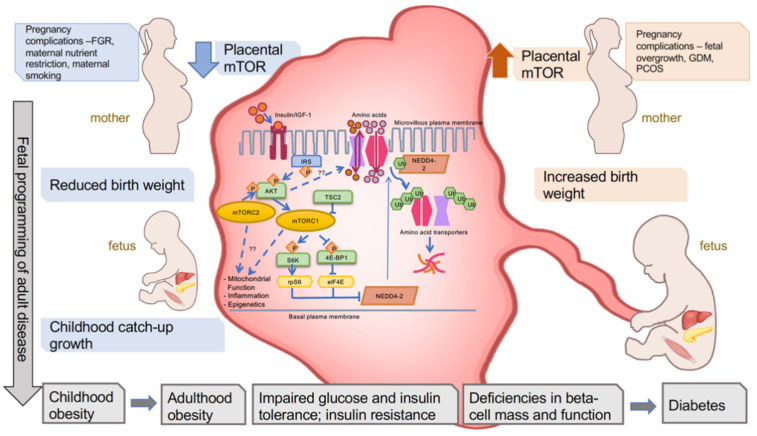
Placental mTOR signaling is altered during pregnancy complications and is associated with birth weight and metabolic health trajectory of the offspring. Various complications during pregnancy lead to decreased mTOR signaling or increased mTOR signaling in the placenta. Upstream regulators and downstream targets of mTOR signaling are depicted, including well-known (solid arrow) and proposed (dashed arrow) players. Decreased placental mTOR signaling and amino acid transport are associated with reduced birth weight and subsequent childhood catch-up growth. Increased placental mTOR signaling and nutrient transport are associated with increased birth weight. There is a positive relationship between both reduced and increased birth weight and childhood obesity, followed by metabolic dysfunction and diabetes in adulthood.

Mechanistic target of rapamycin (mTOR) is a serine/threonine kinase that serves as a core component of mTOR complex 1 and mTOR complex 2. mTOR is activated by growth receptors, such as insulin growth factor (IGF) and insulin receptors, via IRS and the PI3K cascade. Direct downstream targets phosphorylated by mTOR include ribosomal protein S6/S6K1 and 4E-BP1, both involved in protein synthesis, ULK1, which inhibits autophagy, and others (see Figure 1). Newer studies suggest that mTOR complexes may also play a role in responding to and modulating mitochondrial function, inflammation pathways, and epigenetic processes [3,4,5]. With its wide range of cellular targets and effects, mTOR functions as a key regulator of cell metabolism, proliferation, growth, and mobility. In tissues throughout the body, mTOR signaling creates a positive feedback loop to promote glucose uptake into cells, and additionally positively regulates amino acid transporters [6]. Through these mechanisms, mTOR regulates organ growth and size. Therefore, mTOR signaling and related amino acid transport regulated by ubiquitin ligase Nedd4-2 in the placenta play a key role in determining offspring birth weight [7] (see Figure 1).

Maternal-placental-fetal units act in coordination to meet the needs of the fetus and to support the mother simultaneously. Within this coordinated system, mTOR senses and integrates nutrient signals [4,8]. As such, placental mTOR signaling can be altered in response to several environmental and pregnancy-related factors including maternal obesity, nutrient restriction, gestational diabetes mellitus (GDM), preeclampsia (PE), polycystic ovarian syndrome (PCOS) and maternal smoking. Human studies as well as animal models recapitulating environmental intrauterine insults and subsequent placental mTOR perturbations suggest an important link between placental mTOR, birth weight, and the trajectory of offspring metabolic health. Notably, reduced mTOR activity in placentas of fetal growth-restricted babies has been observed in humans [9]; whereas increased placental mTOR activity has been associated with fetal overgrowth [10]. Maternal obesity is linked to several pregnancy complications, such as GDM, PCOS and PE, and increases risk for offspring metabolic dysfunction [11,12,13]. GDM is characterized by insulin resistance caused by insufficient insulin production during later stages of pregnancy. In individuals with PCOS, hormones such as estrogen and androgen are out of balance, often resulting in low estrogen and high androgen levels. Additionally, PCOS symptoms are linked to increased blood glucose and insulin levels, which can increase the production of androgen. Elevated insulin level can also lead to weight gain in PCOS [14]. Preeclampsia is a condition defined by maternal hypertension during pregnancy. Because of the molecular complexity of maternal obesity and associated health complications, babies can be fetal growth-restricted (FGR) or large-for-gestational-age. Several pieces of evidence indicate that birth weight predicts risk for obesity and insulin resistance in childhood, adulthood and beyond [15,16,17]. Various preclinical and human studies have investigated the potential role of placental mTOR in models of maternal obesity and other conditions of placental insufficiency. Association relationships and direct manipulation of mTOR signaling have delineated a strong link between placental mTOR, birth weight, and programming of offspring metabolic health. Moreover, there is evidence that sexual dimorphism is present in offspring metabolic outcomes [18].

This narrative review will focus on the relationship between alterations in placental mTOR signaling associated with intrauterine complications and offspring metabolic health. First, placental mTOR signaling and associated birth weight outcomes will be reviewed for each pregnancy complication, with a special focus on sexual dimorphism within each pregnancy complication if data are available. Subsequently, the offspring metabolic health trajectory will be evaluated through discussions of obesity, insulin sensitivity, and pancreatic beta-cell mass and function. Human pathophysiology is reviewed with discussion of important animal studies to better facilitate readers’ understanding.

### 1.1. Fetal Growth Restriction

Fetal growth restriction (FGR) is defined as fetal weight below the 10th percentile for gestational age. FGR affects up to 10% of all pregnancies in the United States and carries an increased risk of perinatal mortality and morbidity. Long-term FGR complications include higher risk for hypertension, cardiovascular disease and type II diabetes [19]. FGR involves complex misregulation of a variety of placental signaling pathways, including the mTOR-mediated nutrient sensing pathway.

#### 1.1.1. Placental mTOR

In human pregnancies, mTOR activity has been shown to be decreased in placentas of FGR/growth-restricted babies [9]. Altered nutrient transfer (i.e., decreased in FGR and increased in fetal overgrowth) in response to placental insufficiency or perturbation is thought to be modulated by mTOR nutrient sensing. In human FGR placentas, robust decrease in phosphorylated mTOR, increase in phosphorylated p70 (downstream of mTOR and upstream of S6), and decrease in phosphorylated 4E-BP1 (mTOR target) have been observed [20]. Additionally, other growth factor-mediated pathways, including Erk and Akt, are altered; phospho-Erk is significantly increased and phospho-Akt is significantly decreased in FGR placentas [20]. These findings suggest impaired mTOR signaling may directly or indirectly disrupt placental cell growth pathways. Interestingly, placental mTOR protein levels have been correlated with early catch-up growth following FGR [21], which in turn may link mTOR-associated FGR to adverse metabolic outcomes, such as obesity, in childhood and beyond. Direct genetic deletion of mTOR from the placental trophoblasts of mice conferred a significant reduction in newborn body weight that manifested in sexually dimorphic outcomes in insulin resistance and obesity in the adult offspring [18].

#### 1.1.2. Sexual Dimorphism in FGR

FGR itself is biased toward males in the human context [22]. In fact, mTOR, including placental gene expression, is regulated by the mammalian stress response in a sexually dimorphic manner [23]. In a murine model of direct placental mTOR manipulation, genetic deletion of mTOR in the placenta resulted in reduced placental, embryonic, and newborn body weights in female offspring. However, placental mTOR knockout male offspring displayed no differences in placental or body weights [18]. Sexual dimorphism discrepancies between humans and rodent models highlight the true complexity of placental biology in both mammalian systems. In addition, the totality of molecular changes underlying human FGR may not be recapitulated in the context of direct mTOR deletion.

### 1.2. Fetal Overgrowth

Maternal obesity during pregnancy elevates the risk of pregnancy complications and large-for-gestational-age babies [24]. Situations of fetal overgrowth are typically a result of over-nutrition (i.e., maternal obesity) and consequent increase in nutrient transfer from mother to fetus. A recent study assessing nutrient transport in a mouse model of maternal obesity (i.e., high body mass index, BMI) during pregnancy indicates that indeed certain glucose and amino acid transporters are elevated in the placenta, complemented by increased nutrient transport capacity [25]. Additional studies concluded that obese dams displayed increased activation of placental mTOR signaling [26], which may link mTOR nutrient sensing to stimulation of amino acid transport and fetal overgrowth.

#### 1.2.1. Placental mTOR

A Swedish study revealed a positive correlation between maternal BMI during the first trimester of pregnancy, birth weight, and placental insulin and mTOR signaling. These findings were accompanied by an inverse correlation between birth weight and AMPK signaling [10]. These data support the role of placental mTOR signaling in the relationship between maternal obesity and fetal overgrowth. Furthermore, mechanism-driven rodent studies suggest that dams fed a high-fat/high-sugar diet give rise to placentas with enhanced mTOR signaling (determined by S6 and 4E-BP1) and insulin signaling (determined by IRS1 and AKT). Apoptosis and inflammation-related proteins were unaltered [26].

#### 1.2.2. Sexual Dimorphism in Fetal Overgrowth

Male sex has been defined as an independent risk factor for macrosomia or fetal overgrowth in humans [27], perhaps due to male fetuses’ quicker rate of growth. Throughout human pregnancy, males measure larger and have more severe responses to maternal morbidities [28]. Birth weight can be predicted by placental weight. However, Ouyang et al., demonstrated in 2013 that, in term and preterm births, placental weight was not different between males and females, but birth weight was significantly increased for males [29]. In a mouse model, high-fat diet (HFD) feeding during pregnancy affected the placental and fetal weights of male and female offspring. Placental weights were significantly increased in male and female offspring on HFD compared to a control diet [30]. Placental weight also differed by sex, with male placentas being significantly larger under control and HFD conditions compared to female placentas. Interestingly, fetal weights did not differ by sex or by diet in this study [30]. Upregulation of mTOR activity by genetically deleting TSC2, a negative regulator of mTOR complex 1 (mTORC1), in the mouse placenta resulted in no differences in male or female placental weights or perinatal body weights compared to littermate controls [18]. In contrast to decreasing mTOR in the mouse placenta discussed above, there were no sexual dimorphic effects of increasing mTORC1 activity. These data indicate that hyperactivation of mTORC1 via TSC2 deletion is not sufficient to increase placental, embryonic, or newborn body weights.

### 1.3. Gestational Diabetes Mellitus

mTOR signaling is a key pathway in mediating maternal-fetal nutrient transport as described above [8]. Briefly, the mTOR pathway is responsive to environmental cues such as energy and nutrient availability and responds by activating cellular proliferation and growth pathways. Therefore, its role in sensing and integrating signals for nutrient transfer and subsequent growth of the fetus is particularly interesting in the context of GDM, which is characterized by maternal glucose intolerance and hyperglycemia arising from insufficient insulin production or insulin resistance.

#### 1.3.1. Placental mTOR

A study of human GDM placentas showed increased mTOR signaling as determined by phosphorylated p70S6K (downstream mTOR target) in GDM compared to normal term placentas [31]. Other studies of human GDM placentas display increased phosphorylated mTOR and 4E-BP1 [20] and positive correlations of IGF-1 and mTOR signaling with birth weight of newborns from GDM mothers [32]. Martino et al., 2016 aimed to delineate whether placental signaling and pregnancy outcomes differ between maternal obesity and/or GDM and they found that obese women with GDM had babies with higher estimated fetal weight; however, birth weight was not affected by GDM or BMI [33]. Maternal obesity itself reduced placental mTOR levels, whereas obese women with GDM had increased placental phospo-70S6KB1 (downstream target of mTOR) [33]. A rat model of intrauterine programmed GDM showed impairment in the placental mTOR pathway, whereby phosphorylation of SGK1 (downstream mTOR target) was increased [34]. Across models, the consensus of enhanced mTOR signaling in the GDM placenta is reproducible and widely accepted.

#### 1.3.2. Metformin for GDM Treatment

Metformin treatment for GDM introduces another aspect to placental mTOR regulation during pregnancy. Metformin is an anti-diabetic medication commonly prescribed to women with GDM that targets AMPK and subsequently blunts mTOR signaling. Metformin can be transported into fetal blood circulation. Therefore, mTOR signaling in the placenta and developing fetus can be reduced in response to metformin treatment for GDM. In human studies, metformin-exposed offspring are at increased risk for fetal growth restriction and catch-up growth during mid-childhood [35]. In a murine study investigating the role of maternal high-fat diet (HFD) and metformin in regulation of placental mTOR signaling, Grace et al., found in 2019 that inhibition of mTOR signaling in response to metformin treatment only occurred in the context of maternal HFD [36]. Interestingly, in a preclinical model, metformin administration to pregnant dams throughout gestation led to increased pancreatic progenitors and increased neonatal beta-cell fractions. Metformin treatment of embryonic pancreatic buds resulted in upregulated protein expression of mTOR target phospho-S6 [37]. These data present an intriguing dichotomy of metformin effects in fetal organs.

#### 1.3.3. Sexual Dimorphism in GDM

GDM is associated with higher birth weight and increased risk for macrosomia [38], with 15–45% of GDM pregnancies resulting in macrosomic infants [39]. A population-based study performed in Norway found that placental weights were significantly higher in diabetic pregnancies compared to non-diabetic pregnancies [40]. Interestingly, few GDM studies report on placental or birth weight outcomes by sex. Oken et al., in 2016, demonstrated differences in cord blood hormones (insulin growth factors (IGFs), insulin, leptin and C-peptide) in male infants compared to female infants of GDM mothers, whereby males had generally higher hormone levels with the exception of IGF-1. Male infants had lower IGF-1, whereas females had higher cord blood levels of IGF-1 [41].Together, these data link GDM pregnancies with larger birth weight, larger placental weight, and circulating hormone levels that differ by sex. Evidence of sexual dimorphism related to GDM offspring also indicates that risk of being overweight is increased for boys at 5–7 years old, but not for girls [42]. Another study similarly found that GDM showed a male offspring predominance [43]. These findings suggest a greater sensitivity of male offspring to intrauterine glycemia perturbations. Because of limited preclinical GDM models [44], studies specifically investigating placental mTOR in the link between GDM and sexual dimorphic offspring outcomes are lacking.

### 1.4. Polycystic Ovary Syndrome

Polycystic ovary syndrome (PCOS) is an endocrine disorder that commonly affects women of reproductive age. Obesity and insulin resistance commonly characterize PCOS and can present PCOS mothers with an increased risk for GDM and hypertension [14]. PCOS mothers have significantly smaller newborns and show a significantly higher prevalence of smaller-for-gestational age infants [45].

#### 1.4.1. Placental mTOR

Limited human studies have assessed the direct relationship between placental mTOR, PCOS and pregnancy outcomes. One study addressed whether mTOR signaling was increased in PCOS placentas from uncomplicated pregnancies. However, this study found no effect on mTOR signaling in the PCOS placenta versus controls [46]. In a study of rhesus macaques, testosterone treatment before menarche (to recapitulate hyperandrogenemia that characterizes PCOS) was associated with increased placental mTOR signaling during pregnancy [47]. Testosterone combined with Western diet led to 90% of animals developing polycystic ovaries [48]. Women with PCOS have increased risk for poor pregnancy outcomes including restricted fetal growth and fetal overgrowth. Findings from human and animal models may indicate that perturbation in mTOR signaling, specifically, is required for adverse pregnancy outcomes.

#### 1.4.2. Sexual Dimorphism in PCOS

Human and preclinical studies segregating results by sex are lacking in PCOS.

### 1.5. Maternal Nutrient Restriction

Maternal nutrient restriction (MNR) is a phenomenon leading to placental insufficiency and FGR. MNR captures both low protein diets and calorie restriction. Perhaps the most striking evidence linking human MNR to placental insufficiency is the 1944–1945 Dutch Famine, where severe calorie restriction during critical periods of pregnancy resulted in low birth weights and reduced placenta masses [49].

#### 1.5.1. Placental mTOR

In a baboon model, MNR reduced fetal weights by 13%. This reduction in weight was accompanied by a decrease in placental mTOR signaling (S6K, ribosomal protein S6, 4E-BP1) and AKT/insulin signaling (IRS-1, AKT, ERK) as determined by Western blot analysis [50]. A rat study revealed that pregnant rats on a low protein diet (LP, 4% kcal from protein) compared to pregnant rats on a control diet (18% kcal from protein) have reduced placental mTOR signaling, as determined by the lesser phosphorylation of 4E-BP1 and ribosomal protein S6. In later gestation, AKT signaling upstream of mTORC1, was also decreased in LP placentas [51].

#### 1.5.2. Sexual Dimorphism in MNR

Roseboom et al., 2011 reported sexual dimorphic effects in severity of reduction of placental size and area in the Dutch Famine cohort. The effect of MNR during the Dutch Famine on the placental area was more severe for male offspring compared to female [52]. Preclinical models of maternal low protein diets during gestation did not result in differences in newborn offspring body weight by sex [53]. A maternal low protein diet during only the last week of gestation resulted in reduced newborn body weight but was not segregated by sex [54].

### 1.6. Preeclampsia

Preeclampsia (PE) is a hypertensive disorder that occurs during pregnancy. Human studies have presented mixed results surrounding the relationship between PE and birth weight.

#### 1.6.1. Placental mTOR

Human placentas from pregnancies complicated by PE exhibit a mild decrease in phosphorylated mTOR but an increase in downstream phosphorylated 4E-BP1 [20]. A significant increase phosphorylated AKT was observed in PE placentas [20]. Expression levels of upstream regulators (ELABELA, ROR1) of cell growth, proliferation, and presumably the PI3K/AKT/mTOR pathway were downregulated in the PE placenta compared to normal pregnancy placentas [55,56]. As such, trophoblast cell lines with silenced upstream regulators had decreased phosphorylated mTOR and AKT, which recapitulated signaling in the PE placenta [55,56]. Another study analyzing mTOR and amino acid transporters in full-term and pre-term normal placentas as well as PE and FGR placentas found that placental syncytiotrophoblasts have increased protein level of mTOR in conditions of limited nutrient availability (i.e., PE or FGR) [57]. The variability of findings regarding mTOR signaling activity in the human PE placenta suggests that mTOR is altered but many factors may dictate the magnitude and direction of regulation. To accompany this complexity, PE demonstrates variable effects on offspring birth weight, whereby a range of human studies have reported low birth weight and high birth weight outcomes as well as no difference compared to non-PE mothers [58]. Reduced uterine perfusion pressure (RUPP), typically modelled in rats, mimics the insufficient placental perfusion that is characteristic of PE. Using the RUPP model, Akhaphong et al. in 2018 found a non-significant change in mTOR activity markers phospho-S6 and phospho-AKT in placental lysates [59].

#### 1.6.2. Sexual Dimorphism in PE

Mild PE has been associated with normal fetal growth of male offspring and growth restriction of female offspring at neonatal stage [60]. A potential sexual dimorphic effect of RUPP on fetal weight was investigated in Akhaphong et al. in 2018. A significant reduction in fetal weight in both males and females was observed, with no differences in placental weight [59]. In fact, other groups have also demonstrated a reduction in fetal weight and no difference in placental weight in response to RUPP [61,62]. However, another study found that RUPP offspring display decreased fetal weight, increased placental weight, and altered placental zone architecture [63]. Nonetheless, sexual dimorphism has not been detected with regard to placental or fetal body weights in the RUPP model of PE.

### 1.7. Maternal Smoking

The Centers for Disease Control and Prevention estimate that 12% of women smoke during pregnancy in the United States. Maternal smoking is associated with lower birth weight. For example, an analysis of the Tasmanian Infant Health Survey revealed that maternal prenatal smoking was associated with lower placental and birth weight [64]. The Healthy Start cohort based in the United States also concluded that offspring exposure to maternal smoking during the prenatal period had lower birth weight. This study indicated that these low-birth-weight offspring experience rapid BMI growth over the first 3 years of life [65]. Reduced birth weight may be due to altered nutrient supply, reduced uteroplacental blood flow, hypoxia or other placental perturbations caused by chemicals present in ingested cigarette smoke [66]. Nicotine acts as a vasoconstrictor, leading to reduced oxygen supply to the fetus.

#### 1.7.1. Placental mTOR

A preclinical model determined that dams exposed to secondhand smoke for 4 days had offspring with decreased placental and fetal weights (FGR), decreased trophoblast invasion, and decreased activation of placental phospho-mTOR, phospho-p70 and phospho-4E-BP1 [67].

#### 1.7.2. Sexual Dimorphism in Maternal Smoking

Maternal smoking during pregnancy has adverse impacts on fetal programming and offspring health. However, there is a lack of preclinical models of smoking, which limits our understanding of causal factors. In addition, a need for stratification by sex in human investigations and mechanism-driven studies with sex as a biological variable are needed to determine whether there is a sexual dimorphic effect of maternal smoking on offspring metabolic health and resilience.

### 1.8. Relationship between Perturbed Placental mTOR, Birth Weight, and Offspring Obesity

The relationship between birth weight and risk for obesity across the lifespan is well-established. Several findings indicate higher BMI in childhood and adulthood in the offspring of mothers with pregnancy complications. Placental mTOR has been implicated in programming offspring tissues to be more sensitive to a metabolic challenge [18].

#### 1.8.1. Childhood

Observational studies suggest that the offspring of GDM mothers are more likely to develop childhood obesity (or higher BMI) compared to offspring not exposed to maternal hyperglycemia. This finding has been repeated in several studies across the globe [12]. However, intervention studies aimed to provide GDM treatment to mothers did not exhibit differences in offspring childhood BMI. Randomized, controlled trials demonstrated that treatment of mild GDM did not result in large-for-gestational-age babies, nor did the offspring have higher BMI at age 4–5 years [68,69]. Maternal PCOS is also associated with a higher risk of offspring obesity (males and females) from early age and higher diabetes susceptibility in female offspring during adolescence [70].

Several epidemiological studies have reported an association between maternal smoking and childhood/adolescent obesity [66,71], possibly due to the catch-up growth experienced by fetal growth-restricted infants of mothers who smoked during pregnancy [72]. Offspring of smokers displayed higher BMIs than offspring of non-smokers by age 5, and the trajectory continues thereafter. This trend of higher BMI in childhood is especially evident in female offspring [66]. Experimental studies using rodent models of maternal smoking during pregnancy and lactation reveal increased fetal adiposity and increased body weights at 5- and 9-weeks of age [71]. The observation of sexual dimorphic effects of maternal smoking on female offspring obesity in humans is substantiated in rodents, whereby higher weight gain in female offspring was persistent until 90 days of age; however, the effect was transient in male offspring [73].

#### 1.8.2. Adulthood

Young adult males from the Dutch Famine cohort were at greater risk of developing obesity following exposure to nutrient restriction in utero. This finding was specific to enduring severe under-nutrition during first half of pregnancy [74]. Similarly, adult offspring of mothers who smoked during pregnancy had increased incidence of obesity [75]. Offspring of women with PCOS were at higher risk of metabolic disorder [76]. In a PCOS-like mouse model, female offspring had increased body weight postnatally until 12 weeks of age. Male offspring from PCOS-like dams exhibited a mild increase in body weight from birth until 4 weeks, but the body weights are comparable to controls after 4 weeks [77].

Direct mTOR deletion in the placenta resulted in offspring with obesity outcomes that were different between sexes. Neither male nor female offspring with placental mTOR knockout developed obesity when subjected to a normal chow diet (18% kcal from fat) for 12-weeks. However, when metabolically challenged with a high-fat diet (60% kcal from fat), females became more obese as compared to littermate controls, whereas male offspring on a high-fat diet did not develop obesity [18].

### 1.9. Relationship between Placental mTOR, Birth Weight, and Offspring Insulin Sensitivity

Multiple pregnancy complications are associated with reduced placental mTOR signaling and low birth weight. Low birth weight also has a connection to insulin resistance in adulthood. Due to the timescale needed to study the development of insulin resistance in adulthood, there are limited human studies investigating this relationship in a longitudinal manner. However, evidence from the Dutch Famine cohort and preclinical models have substantiated this relationship [78].

#### Adulthood

Individuals from the Dutch Famine cohort have reported low birth weight due to prenatal exposure to under-nutrition. In adulthood, this cohort displays impaired glucose tolerance and insulin resistance possibly related to inadequate insulin secretion or action [78]. Another famine study found that only females exposed to fetal or childhood famine had a significant positive association with insulin resistance, as estimated by the homeostasis model assessment index of insulin resistance (HOMA-IR) [79]. In a preclinical model of low protein exposure throughout pregnancy (LP0.5), both male and female offspring exhibited impaired glucose tolerance and enhanced insulin sensitivity at six weeks of age [53]. A low protein diet only during the last week of murine pregnancy (LP12.5) resulted in male offspring developing high fat diet-induced insulin resistance, whereas females developed insulin resistance by aging [54]. RUPP offspring also have greater HOMA-IR at nine weeks of age [62]. Female offspring of PCOS-like dams had significantly impaired glucose tolerance. However, glucose tolerance of male offspring was unaffected compared to controls. Interestingly, insulin levels were not significantly different among groups (control versus PCOS-like mothers, male versus female offspring) [77].

A 2002 study was the first to link maternal smoking to development of early adult-onset diabetes [75]. Since that time, limited preclinical models of maternal smoking have bolstered these findings. Maternal cigarette smoke exposure is associated with increased level of fasting serum insulin in adult offspring. Neonatal nicotine exposure impaired offspring glucose tolerance in early and late adulthood, which was suggested to be due to reduced peripheral insulin sensitivity [80]. Robust maternal smoking models and direct evidence of delineating mechanisms are needed to determine causality.

Female offspring with placental mTOR knockout in utero displayed high-fat diet-induced insulin intolerance in adulthood, accompanied by hyperinsulinemia. On the other hand, male offspring with placental mTOR knockout had comparable glucose and insulin tolerances to littermate controls [18].

### 1.10. Relationship between Placental mTOR, Birth Weight, and Beta Cell Mass/Function

Determining the relationship between placental mTOR and offspring pancreatic beta-cell mass and function comprises a newer area of research. Direct placental mTOR knockout gives rise to sexually dimorphic effects on beta-cell mass compensation, discussed in more detail below. The relationship between intrauterine insults such as preeclampsia and nutrient restriction, which have previously been associated with altered mTOR signaling in the placenta, have been studied in the RUPP model of preeclampsia and in maternal low-protein diet investigations [53,59].

#### Adulthood

In the gestational hypertension RUPP model, increased beta-cell death, reduced beta-cell area, and decreased mTOR protein level in the offspring fetal pancreas were reported [59]. In response to a maternal low-protein diet during pregnancy (starting at embryonic day 0.5, LP0.5), beta-cell mass of offspring at birth was significantly reduced. However, at 3 months of age, both male and female offspring beta-cell mass was normalized to control. Interestingly, male offspring displayed defects in insulin secretion in adulthood in part by reduced islet mTOR expression level. Islet insulin content was significantly reduced only in female adult offspring [53]. These findings highlight potential sexual dimorphism in programming beta-cell mass and function. While this study assessed mTOR signaling in the beta-cell, it did not address alterations in placental mTOR.

Direct ablation of placental mTOR reduced placental weight in the female offspring but did not alter beta-cell mass in both male and female neonates. Adult female and male offspring showed normal glucose homeostasis under a normal chow diet. While adult female offspring with mTOR knockout in utero demonstrate high-fat diet-induced insulin resistance, they have normal beta-cell mass and function compared to littermate controls [18].

## 2. Biology Underlying Sexual Dimorphism during Fetal Programming

Differences in placental and fetal gene expression, epigenetic mechanisms, and hormone levels have been identified early in fetal development. Dearden et al., in 2018, elegantly reviewed several mechanisms mediating sexual dimorphic programming leading to adult metabolic disruption [81]. Despite the placenta being thought of as a sexless organ, the contribution of sex gene differences (allosomal genes such as O-GlcNAc transferase (OGT)) between males and females has been implicated to mediate the programming of the fetus. For example, estrogen serves as a player in reducing risk for obesity, type II diabetes, and cardiovascular disease through effects on offspring insulin and leptin levels during gestation [82,83]. A critical role for epigenetic programming, which can also be a result of an adverse intrauterine environment, has been proposed as a driving mechanism behind altered fetal programming of certain metabolic tissues. DNA methylation, histone modifications, chromatin remodeling, and microRNAs comprise only a portion of the epigenetic mechanisms modulating transcriptional control of developing fetal tissues. Recent studies have reported a link between DNA methylation machinery and sex hormones [84]. Post-translational modification players, such as X-linked OGT, have been demonstrated to be at lower levels in the male fetus, making males particularly susceptible to the intrauterine environment [85]. Metabolic hormones such as leptin and insulin also have effects on the developing fetus. Higher levels of leptin have been detected in female cord blood compared to male cord blood [86]. Circulating leptin at a neonatal age can come from the placenta, the fetus, or the mother. Glucose and insulin levels of neonates differ during gestation and at birth.

## 3. Conclusions and Future Directions

The importance of mTOR signaling in the placenta to regulate fetal development and programming of metabolic organs is gaining momentum. The link between perturbed placental mTOR and adverse birth weight outcomes has been assessed in several pregnancy complications, and a robust relationship between birth weight and offspring metabolic health trajectory has been defined. However, longitudinal studies and mechanistic investigations are lacking in the current body of research. Longitudinal human studies, as well as preclinical studies designed to follow mother and offspring from pre-conception, through gestation, and across the lifespan are needed to draw causal conclusions. Mechanistic studies investigating molecular changes occurring in response to placental mTOR perturbations both locally in the placenta and systemically throughout the developing fetus will be necessary to understand the sexual dimorphic metabolic outcomes observed. In addition to those highlighted in the previous section, several other mechanisms mediating sexual dimorphism include alterations in inflammation, nutrient transport, and hypoxia in the placenta and developing fetal tissues.
